# Sustainable Microwave-Assisted Extraction of Hemp Seed Oil as Functional Additive into Polybutylene Succinate (PBS) Films for Food Packaging

**DOI:** 10.3390/polym17101376

**Published:** 2025-05-16

**Authors:** Giovanni Dal Poggetto, Mattia Di Maro, Luca Gargiulo, Donatella Duraccio, Gabriella Santagata, Giovanna Gomez d’Ayala

**Affiliations:** 1 Institute of Polymers, Composites and Biomaterials (IPCB)-CNR, Via Campi Flegrei 34, 80078 Pozzuoli, Italy; giovanni.dalpoggetto@cnr.it (G.D.P.); luca.gargiulo@cnr.it (L.G.); giovanna.gomezdayala@cnr.it (G.G.d.); 2 Institute of Sciences and Technologies for Sustainable Energy and Mobility (STEMS)-CNR, Strada Delle Cacce 73, 10135 Torino, Italy; mattia.dimaro@stems.cnr.it

**Keywords:** hemp seed oil, microwave assisted extraction, polybutylene succinate (PBS), food packaging, migration, plasticizer effect

## Abstract

In this study, microwave-assisted extraction (MAE) was performed to recover antioxidant hemp seed oil (HSO) with the purpose of developing polybutylene succinate (PBS)/HSO-based films for active packaging to improve food shelf-life. It was found that MAE achieved comparable yields, structural characteristics, and antioxidant activity to Soxhlet extraction, but in significantly less time (2.5 min vs. 6 h). PBS-based films with 0.5 and 1 wt% HSO were prepared by compression molding. Morphological investigation of the PBS-HSO films highlighted uniform oil droplet dispersion and good compatibility. HSO reduced PBS crystallinity but did not affect the α-form of PBS. Thermal analysis showed reductions in T_m_ and T_c_, whereas T_g_ remained unchanged at −17 °C. PBS containing 1 wt% HSO exhibited a 42% decrease in Young’s modulus, 47% reduction in elongation at break, and 47% decrease in tensile strength due to the plasticizing effect of the oil and, which reduced the intermolecular forces and facilitated polymer chain disentanglement, in agreement with the FTIR analysis, which showed a distinct broadening of the carbonyl stretching region associated with the amorphous phase (1720–1730 cm^−1^) in the PBS-HSO films compared to neat PBS. Migration tests showed that the films are unsuitable for fatty foods but safe for aqueous, acidic, and alcoholic foods.

## 1. Introduction

The growing global concern about environmental pollution, especially due to plastic waste, has underlined the need for sustainable alternatives to conventional petroleum-based packaging materials [1,2]. Traditional petroleum-based plastics, such as polyethylene (PE) and polypropylene (PP), which are commonly used in food packaging [3,4,5], are non-biodegradable, posing significant challenges in terms of waste management and recycling [3]. As an alternative, biodegradable bio-based polymers could represent a promising solution due to their ability to degrade naturally in the environment. Among them, polybutylene succinate (PBS) stands out for its good mechanical strength, thermal stability, and biodegradability, making it a promising material for food packaging applications [6,7]. However, to fully unlock its potential exploitation, particularly in flexible packaging, improvements in its plasticization and toughness are still needed. To overcome these drawbacks, plasticizers are commonly employed. These additives are commonly used to improve the flexibility and processability of biopolymers [8,9]. Anyway, since conventional plasticizers are sourced from petroleum, there is growing interest in using natural, renewable, bio-based alternatives. In particular, plasticizers derived from waste biomass and industrial byproducts present a sustainable solution aligned with the principles of a circular, zero-waste bioeconomy [10,11,12]. In this context, hemp (*Cannabis sativa* L.) seeds that are unsuitable for the food market can serve as an excellent, low-cost source of oil, which can be used both as a plasticizer and as a source of antioxidant compounds [13]. Hemp seed oil (HSO) is known for its multiple beneficial properties, including anti-inflammatory, antioxidant, and antibiofilm effects [14]. Moreover, it boasts a rich nutritional profile, primarily due to its high content of polyunsaturated essential fatty acids (EFAs) such as linoleic acid (C18:2ω6) and α-linolenic acid (C18:2ω3) [15].

The oil content in hemp seeds varies from 28% to 35%, depending on the variety, climatic conditions, geographic region, and year of growing cultivation [16]. Besides oil, hemp seeds also contains 20–25% protein, 20–30% carbohydrates, 10–15% fiber, and trace amounts of minerals [17].

HSO is traditionally extracted using cold pressing [16,18] or solvent-based methods [19]. Among these, Soxhlet (SOT) extraction is commonly used due to its relatively low cost and high oil yield. Nevertheless, it requires extended extraction times and the use of chemical solvents, leading to hazardous conditions for both workers and the environment [20]. By contrast, microwave-assisted extraction (MAE) has emerged as a more sustainable and efficient extraction method [21,22]. Indeed, according to the literature, microwave radiation significantly accelerates the extraction process, leading to higher yields, solvent reduction, and lower energy consumption [23,24,25]. In this context, this study aims to achieve two primary goals. Firstly, microwave-assisted and Soxhlet extractions applied to a hemp seeds/n-hexane system were compared in order to assess the sustainability of the MAE method. Subsequently, the HSO obtained through MAE was incorporated into a poly (butylene succinate) (PBS) polymer matrix to develop bioactive packaging materials. The obtained PBS/HSO films were thermally and structurally characterized. Finally, their antioxidant activity and interactions with food simulants were carried out as preliminary data for potential future application.

## 2. Materials and Methods

### 2.1. Hemp Seed Pre-Treatment

The hemp seeds used in this study were sourced from the “Carmagnola” variety of hemp (*Cannabis sativa* L.) grown in the Piedmont region of northwestern Italy. Initially, the seeds were sieved and thoroughly washed with water to eliminate any soil particles. After drying, the seeds were finely ground in a laboratory mill under cryogenic conditions using liquid nitrogen. The resulting powder was then subjected to extractions using both Soxhlet (SOT) and microwave-assisted extraction (MAE) methods.

### 2.2. Soxhlet and Microwave-Assisted Extractions

The Soxhlet extraction of the ground hemp seeds was performed dispersing 10 g of seeds in 200 mL of n-hexane. The mixture was refluxed for 6 h at 70 °C, the boiling point of n-hexane, followed by solvent removal under vacuum using a rotary evaporator (IKA RV 10 basic) until a constant weight was achieved.

The MAE experiments were carried out using a Microwave 5000 (Anton Paar, Graz Austria) following a solid–liquid extraction method. The extractions were performed at different irradiation times—2.5, 5, 15, 30 min, and 1 h—using a power setting of 300 W, a temperature of 50 °C, and n-hexane as the solvent. The microwave system consisted of 16 vessels, each filled with 2 g of hemp seed powder and, additionally, silicon carbide cylinder was inserted as a heat transmitters. The ground seeds/solvent ratio was 1:10 (i.e., 2 g of powdered hemp in 20 mL of n-hexane for each vessel). After each irradiation, the seed powder and solvent were allowed to cool at room temperature and were centrifuged at 10.000 rpm for 30 min at 4 °C. The extract underwent solvent removal utilizing a rotary evaporator until complete dryness was achieved. Subsequently, the extracts were subjected to vacuum conditions until reaching a consistent weight.

For both the Soxhlet and MAE extractions, the obtained oils were weighed, and the extraction yield was calculated using the following equation:

(1) $$\gamma \;(\%)=\frac{M_{oil(g)}}{M_{mass\;of\;hemp\;seed\;(g)}}\times 100$$

Each experiment was performed in duplicate.

Based on the results of the MAE oil extraction, in terms of yield and oil structural and functional properties, detailed in the Section 3 of the Results and Discussion, the lowest irradiation time of 2.5 min was selected as the most suitable and eco-cost effective one.

### 2.3. PBS-Hemp Oil Blend and Film Preparation

Blends of PBS and selected HSO were prepared by solvent casting. PBS, supplied by Novamont (Novara, Italy) and used for producing packaging films, is a carboxy-terminated polymer (M_w_ ~10^5^ g/mol with a content of COOH terminal groups of 77 m_eq_/kg). PBS and hemp oil were separately dissolved in chloroform at specific concentrations. Specifically, three different formulations were prepared using different amounts of HSO, namely, 0.5, 1, and 2.5 wt% with respect to the polymer matrix mass. This concentration range was selected accordingly to a previous study in order to ensure a homogeneous oil distribution without noticeable phase separation or oil exudation [26]. By comparison, neat PBS solution was also prepared. Subsequently, the solutions were combined and stirred for 1 h to facilitate the dispersion of the oil within the polymer matrix. The resulting mixture was then dried using a rotary evaporator to eliminate all traces of solvent. Hereinafter, the PBS blends containing oil extracted by MAE for an irradiation time of 2.5 min in the amounts of 0.5, 1, and 2.5 wt% are coded as PBS-HSO0.5, PBS-HSO1, and PBS-HSO2.5, respectively. The blends were compression-molded employing a hydraulic press (Collin P200T, Ebersberg, Germany). Both neat PBS and the PBS/oil blends were heated to 140 °C for the first 4 min without applied pressure and for another 4 min with the pressure raised to 50 bar. Subsequently, the samples were rapidly water-cooled to room temperature. Before processing, the PBS powder was dried in an oven at 60 °C under vacuum for 24 h to avoid hydrolytic degradation phenomena. Films with a thickness of approximately 120 µm were obtained as a result.

### 2.4. Hemp Seed Extract and Film Characterization

#### 2.4.1. Spectroscopic Analyses (FTIR-ATR)

Fourier-transform infrared spectroscopy (FTIR) analysis was carried out in attenuated total reflection (ATR) mode using a Perkin-Elmer Spectrum 100 spectrometer (Waltham, MA, USA). The spectra (32 scans) were recorded in the 4000–400 cm^−1^ wavelength range at 4 cm^−1^ resolution. Spectroscopic manipulation was performed with OMNIC 9 software (Thermo Fisher Scientific, Inc., Waltham, MA, USA). Both hemp seed extracts and films were characterized by ATR.

#### 2.4.2. DPPH Radical Scavenging Activity

In the DPPH assay, the antioxidant activity is based on the inhibition of the DPPH radical (2,2-diphenyl-1-pi-crylhydrazyl) by the antioxidants (Brand-Williams, Cuvelier, & Berset, 1995). The extracted oils were diluted to a final concentration of 400 µg/mL in ethanol. Subsequently, 1 mL of ethanol and DPPH radical solution (0.6 mM) was added to 3 mL of the solution containing the sample. The mixture was agitated by vortexing and brought to room temperature in the dark for 30 min. The absorbance at 517 nm was then measured and the percentage inhibition, also indicated as % DPPH, was calculated according to the following formula:

(2) $$\%\;inhibition\;(\%\;DPPH)=\frac{\left(A_{c}\right)-\left(A_{s}\right)}{\left(A_{c}\right)}$$

where A_c_ is absorbance of the control and A_s_ is the absorbance of the tested sample. Ethanol (1 mL) was used as a white, while ethanol and DPPH (1.0 mL; 0.6 mM) and ascorbic acid were used as negative and positive controls, respectively.

#### 2.4.3. Wide Angle X-Ray Diffraction (WAXD)

WAXD of neat PBS and its HSO blends was performed in Bragg-Brentano geometry using a PW3040/60 X’Pert PRO MPD diffractometer (PANalytical, Eindhoven, The Netherlands) operating at 45 kV and 40 mA. The X-ray source was a high-power ceramic tube PW3373/10LFF with a Cu anode, emitting Ni-filtered Cu-Kα radiation (λ = 0.15418 nm). The WAXD profiles were acquired in the range 5–60°, with continuous scanning at 0.04°/s. The crystallinity degree of the samples was calculated by the ratio between the area corresponding to the crystalline fraction of PBS and the total area of the diffraction spectrum. The area of the crystalline fraction corresponded to the total area of the spectrum from which the contribution to scattering due to the amorphous phase of the polymer was subtracted. The spectrum of the amorphous phase of PBS was estimated using Origin Lab 8.5 software, building a baseline underlying the XRD spectrum with a Gaussian shaped curve.

#### 2.4.4. Scanning Electron Microscopy (SEM)

The cross-section of the films was examined via scanning electron microscopy (SEM) employing a ZEISS EVO 50 XVP instrument equipped with a LaB_6_ source (Zeiss Microscopy, Oberkochen, Germany). To observe the cross-section, samples were cryogenically fractured using liquid nitrogen. Before observation, a thin layer of gold (approximately 10 nm) was applied to the samples to prevent any charging effects.

#### 2.4.5. Differential Scanning Calorimetry (DSC)

The thermal properties of PBS, both with and without HSO, were examined via differential scanning calorimetry (DSC) using a DSC Q20 instrument from TA Instruments. Heating was conducted at a rate of 20 °C/min. The experimental procedure comprised the following cycle: (1) heating from 25 °C to 150 °C, (2) cooling to −60 °C, and (3) reheating from −60 °C to 150 °C. The glass transition temperature (T_g_) was determined from the first derivative curve of the second heating thermogram [27]. The crystallinity degree of PBS and the blends was calculated as follows:

(3) $$Xc=\frac{{\Delta H}_{m}}{{\Delta H}_{PBS}^{0}}\;\times \;100$$

where ΔH_m_ is the measured enthalpy of melting and ΔH^0^_PBS_ is the enthalpy of melting per gram of 100% crystalline PBS (110.3 J/g) [28]. Each experiment was conducted using 6.0 ± 0.5 mg of material.

#### 2.4.6. Thermogravimetric Analyses (TGA)

Thermogravimetric analysis was performed using a thermogravimetric analyzer (TGA/DTG Perkin-Elmer Pyris Diamond). Measurements were performed on samples of about 4–6 mg, placed in open ceramic crucibles and heated from room temperature to 600 °C at a heating rate of 10 °C min^−1^ in a nitrogen atmosphere (nominal gas flow rate of 30 mL min^−1^). The onset degradation temperature (T_onset_) was calculated considering the temperature at which a weight loss (WL) of 10 wt% occurred, while the temperature at which the degradation rate reached a maximum (T_peak_) was evaluated as the peak of the first TG derivative (DTG).

#### 2.4.7. Mechanical Properties

Tensile tests were carried out using an Instron dynamometer model 4301 (Canton, OH, USA) equipped with a load cell of 1 kN. The width and the length of the investigated films were 4 and 28 mm, respectively, while the thickness of each film was 0.12 mm. All of the measurements were carried out at 25 °C and 50% relative humidity (RH), at a crosshead rate of 5 mm/min. The Young’s modulus, stress, and strain at break points were determined. All of the mechanical analyses were performed on five specimens previously conditioned at room temperature and at a relative humidity equal to 50%.

#### 2.4.8. Migration Tests in Food Simulants

In this work, EU Regulation No. 10/2011 concerning plastic materials and articles intended to come into contact with food was considered for performing migration tests. Three simulants containing different concentrations of ethanol were used: 10% (simulant A), 20% (simulant C), and 50% (simulant D1). Simulants A and C were chosen for foods with hydrophilic properties, covering aqueous, acidic, and alcoholic foods. Specifically, simulant A is assigned for foods like fruits, vegetables, eggs, meat, and fish; simulant C was assigned for foods like coffee, syrups, or jams. Instead, simulant D1 was chosen for foods that have lipophilic properties. PBS blend specimens of 1 dm × 1 dm were dried in an oven at 40 °C overnight and weighed. After, they were immersed in 100 mL of simulant solution at 40 °C for 10 days [29]. After these days, the samples were removed from the solutions and dried in an oven at 40 °C and weighed again. The percentage of release (or migration) from the PBS food contact material into the food simulant was measured by comparing the amount of migrant into the simulant with the film initial weight. The general formula is:

(4) $$\%\;release=\frac{w_{t}-w_{t0}}{w_{t0}}\times 100$$

where *w_t_* is the film weight at time t and *w_t_*_0_ is the film weight at the beginning of the test (t = t0).

## 3. Results and Discussion

### 3.1. Extract Characterization

Microwave-assisted extraction (MAE) is a fast and efficient technique in which microwave energy uniformly heats the biomass, enhancing the diffusion of compounds into the solvent. For this reason, it is a much more effective method if compared with the classical Soxhlet extraction [30,31], often completing extractions within minutes due to the rapid heating provided by microwave energy. In light of this, the extraction yields of MAE and SOT procedures applied to a hemp seeds/n-hexane system were compared (Table 1). SOT extraction after 6 h produced a yield of 30%. All MAE extractions exhibited yields comparable to that obtained with Soxhlet. This result highlighted the efficiency of the MAE process, which achieved yields comparable to the 6 h Soxhlet extraction in only 2.5 min.

#### 3.1.1. DPPH Assay

In addition to its nutritional value, HSO is also rich in natural antioxidants, such as phenolic compounds, tocopherols, and phytosterols [32]. The DPPH assay was performed to confirm the antioxidant properties of the extracts, and the obtained results are listed in Table 1. The DPPH values of oil extracted by MAE were very close to each other and comparable with that of the SOT method, indicating that by MAE it is possible to obtain oils with properties comparable to those obtained by traditional methods but with greatly reduced operating time.

To verify that the conditions of the film preparation process (compression molding at 140 °C) did not compromise HSO bioactivity, the oils were subjected to a heating treatment through TGA in oxygen, using a temperature ramp from 25 °C up to 140 °C at 10 °C/min and then kept at this temperature for 20 min. The DPPH values of all of the extracted oils did not change after the thermal treatment, confirming their suitability for use as functional additives for PBS.

#### 3.1.2. FTIR-ATR Spectroscopy of HSO and of PBS-Based Films

**Figure 1:**
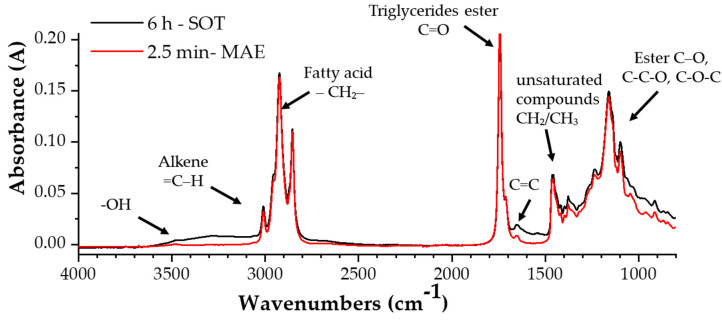
FTIR-ATR spectra of hemp seed oil obtained with different extraction procedures and at different times (SOT for 6 h and MAE for 2.5 min), in the region between 4000 and 600 cm^−1^.

Spectroscopic analysis was performed to identify and characterize the functional groups of the extracted HSO and to highlight the likely structural differences between SOT and MAE oils. The analysis provided insights into the molecular composition and structural features of the oils. In Figure 1, for the sake of clarity, only the FTIR-ATR spectra of SOT and MAE2.5 are reported, since overlapping spectra were observed for the oils extracted by different MAE irradiation times. The characteristic peaks attributable to fatty acids were found at 2926 cm^−1^ and 2855 cm^−1^, corresponding to the asymmetric and symmetric stretching vibrations of methylene (–CH_2_–) groups in aliphatic chains. The weak and sharp band at 3011 cm^−1^ was characteristic of the =C–H stretching vibration in alkenes (C=C–H), i.e., double bonds with vinylic hydrogen atoms. It suggested the presence of unsaturation in lipids, such as oleic, linoleic, or linolenic acids, all commonly found in HSO [33].

The overlapped bands at 1745 cm^−1^ were attributed to the C=O stretching vibration of ester functional groups commonly found in triglycerides, whereas the shoulder at 1715 cm^−1^ was likely due to the presence of free fatty acids or carboxylic acids, possibly resulting from partial hydrolysis of triglycerides during extraction. A broad and weak absorption band centered around 3400 cm^−1^ was observed exclusively in the Soxhlet-extracted oil, but not in the MAE extract. This feature was typically attributed to -OH stretching vibrations of hydrogen-bonded hydroxyl groups and was most likely due to the presence of residual water retained during the Soxhlet extraction process.

Since both extracts were obtained from the same hemp biomass, and no such band was detected in the MAE spectrum, the contribution of hydroxyl-containing cannabinoids (e.g., phenolic O–H groups) could be reasonably excluded. Therefore, the absence of the 3300–3400 cm^−1^ region band in the MAE extract supported the conclusion that cannabinoids were not present in detectable amounts in either extract. Additional peaks at 1657 cm^−1^ and 1463 cm^−1^ may corresponded to C=C stretching and CH_2_/CH_3_ bending vibrations, respectively, and were consistent with the presence of unsaturated compounds, such as carotenoids. In the fingerprint region, additional vibrational modes at 1241 cm^−1^, 1164 cm^−1^, and 1100 cm^−1^ were assigned to C–O, C-C-O, and C-O-C stretching and bending vibrations of ester functionalities, further confirming the presence of glycerol-based lipids such as mono-, di-, or triglycerides in the oil matrix. Finally, the peak at 722 cm^−1^ was associated with the rocking vibrations of long aliphatic –CH_2_– chains, typical of fatty acids [34].

The results of the oil characterization confirmed that MAE extraction was a much faster method if compared with the classical SOT extraction and that after 2.5 min it was already possible to obtain the same yield (see Yield (%) in Table 1), the same antioxidant properties (see DPPH assays in Table 1), and the same structural properties, as previously confirmed by spectroscopic analysis of SOT- and MAE-extracted oils. For these reasons, MAE-extracted oil in 2.5 min was used for preparing blend films, as described in Section 3.2.

### 3.2. PBS-Oil Blend Film Characterization

The extracted oil, previously characterized in terms of functional and structural properties, were incorporated into PBS at different concentrations (0.5, 1, and 2.5 wt% with respect to PBS mass) for the development of PBS-based films via compression molding. This process was carried out under conditions designed to preserve the bioactivity of the functional compounds, as demonstrated by the DPPH assay performed on HSO subjected to the same heat treatment by TGA, and resulted in homogeneous films with a thickness of 120 µm. It is worthwhile to underline that the blend containing 2.5 wt% oil exhibited an immediate post-processing oil exudation. The oil migrated to the film surface forming visible droplets. Such behavior is commonly observed in polymer-based systems loaded with oils or plasticizers in which, over time, phase separation and surface migration may occur [35]. This exudation can lead to surface contamination, changes in mechanical properties, and aesthetic defects. For this reason, the 2.5 wt% formulation was not considered for further characterization.

#### 3.2.1. Structural Analysis (XRD)

**Figure 2:**
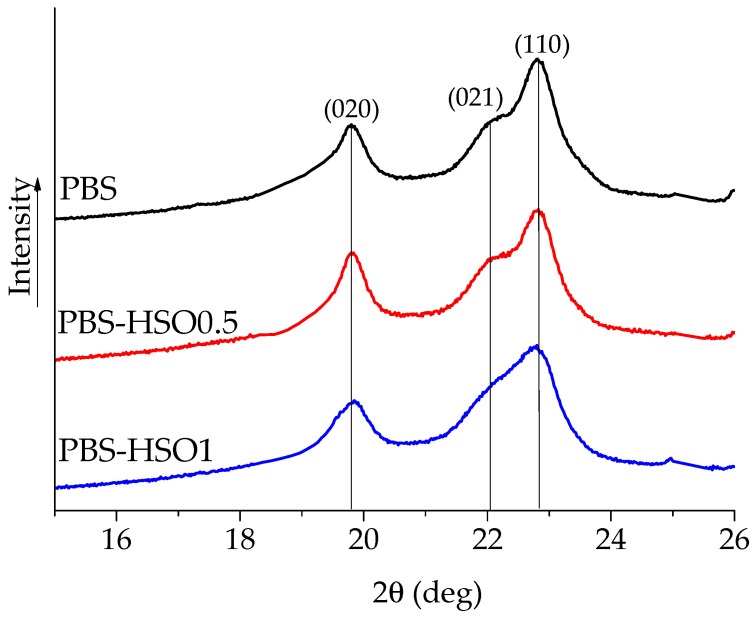
XRD spectra of films of PBS and PBS-HSO blends containing different amounts of hemp seed oil extracted by MAE.

The XRD profiles of the neat PBS film and the blends are reported in Figure 2. The neat PBS film spectrum presented the characteristic diffraction peaks of the α-form at 19.8°, 22.0°, and 22.8° related to the (020)_PBS_, (021)_PBS_, and (110)_PBS_ planes, respectively [36]. The position of the α-form peaks remained unchanged in the presence of the oil, whereas the shape for the PBS-HSO1 blend became broader, indicating distortions or lattice strain (variations in d-spacing across the crystal) due to the presence of the oil. The crystallinity degree of neat PBS and its films is reported in Table 2. The commercial PBS film showed a crystallinity degree of 51%; the addition of HSO led to a decrease in the degree of crystallinity, which became more pronounced as the oil content increased. Specifically, PBS-HSO0.5 showed a crystallinity degree of 45% and PBS-HSO1 showed a crystallinity degree of 43%. This effect, already reported in the literature [37,38], is due to the presence of oil, which prevents the PBS chains from organizing and crystallizing as easily as in the neat polymer.

#### 3.2.2. Scanning Electron Microscopy (SEM)

**Figure 3:**
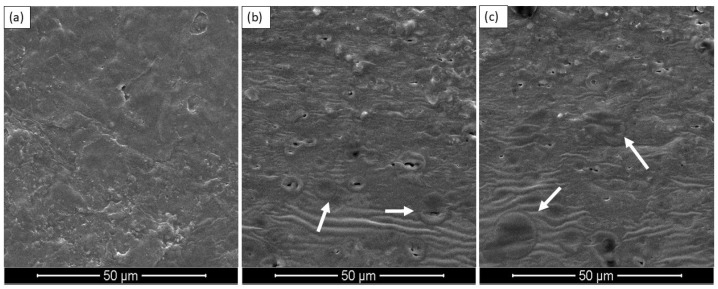
SEM micrographs of (**a**) neat PBS, (**b**) PBS-HSO0.5, and (**c**) PBS-HSO1 film cross-sections.

SEM micrographs of the cross-section surfaces of PBS-based films are reported in Figure 3. Neat PBS (Figure 3a) revealed a smooth and homogeneous surface. By contrast, the PBS-HSO0.5 (Figure 3b) and PBS-HSO1 (Figure 3c) films displayed circular features (with arrows) with diameters varying in the range between 5 and 8 µm, which were not present in the neat PBS sample. They were attributed to oil micro-droplets dispersed within the polymer matrix. Their good embedding inside the delaminated fracture surface and homogeneous distribution throughout the material suggested good interfacial compatibility with PBS. Finally, some dark voids were detectable on delaminated surfaces; they were likely due to oil evolution following cryogenic fracture.

#### 3.2.3. FTIR-ATR Spectroscopy of PBS-Based Films

**Figure 4:**
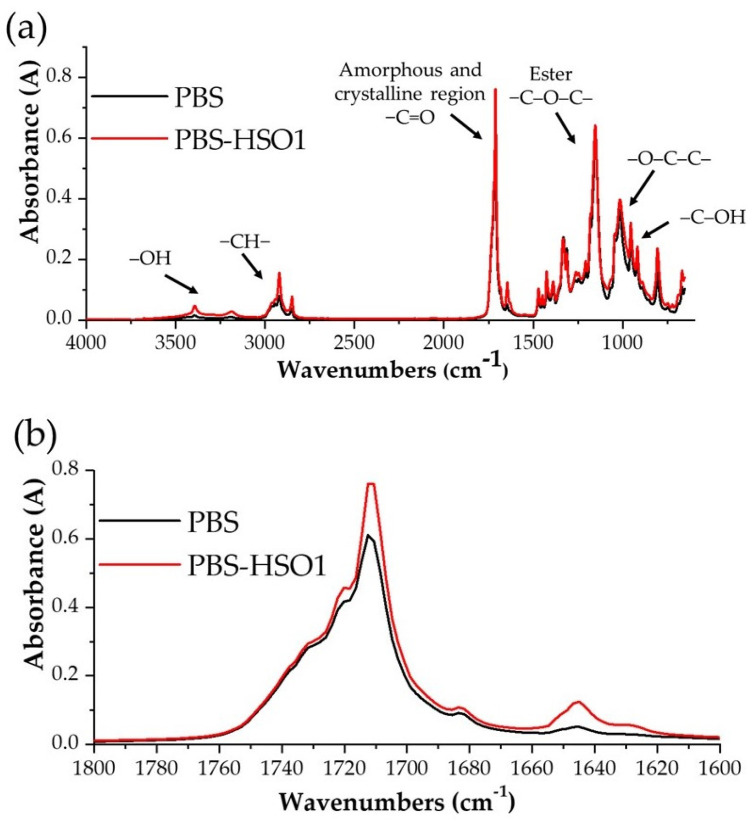
FTIR-ATR analysis of PBS and PBS-HSO1: Spectra in the region 4000 and 650 cm^−1^ (**a**) and in the carbonyl region between 2000 and 600 cm^−1^ (**b**).

Infrared spectroscopy was employed to investigate the primary functional groups of the pristine polyester and to gather insights into the potential interactions occurring after the melt blending process with HSO. To not overburden the FTIR spectrum and to better evidence the spectral differences, only PBS and PBS-HSO1 spectra are reported and overlapped in Figure 4a. A closed view of the carbonyl region is reported in Figure 4b. Regarding PBS, several characteristic absorptions were identified: the very weak and sharp band around 3678 cm^−1^ typically attributed to the free stretching vibration of terminal hydroxyl (–OH) groups indicated the presence of non-hydrogen-bonded chain ends, as expected, since the PBS used was a carboxy-terminated polymer with a content of COOH terminal groups of 77 m_eq_/kg. The absorption band between 3390 and 3400 cm^−1^ was attributed to –OH stretching vibrations weakly involved in hydrogen bonding, possibly related to terminal groups and some absorbed moisture, whereas the peak around 3190 cm^−1^ was attributed to the stretching vibrations of hydroxyl (–OH) groups involved in hydrogen bonding, either between polymer chains or with absorbed moisture. In the range of 3000–2800 cm^−1^, the asymmetric stretching of the –CH– groups was observed, with the corresponding symmetric mode appearing at 1335 cm^−1^.

As typically seen in semicrystalline polyesters, the carbonyl (–C=O) region exhibited multiple overlapping bands. According to previous studies, these arise from the carbonyl stretching vibrations in both the amorphous and crystalline regions of the polymer [39].

For neat PBS, a broad band attributed to the amorphous carbonyl stretching was detected as a shoulder between 1720 and 1730 cm^−1^, adjacent to a sharper, more intense peak at approximately 1711 cm^−1^, which was related to the crystalline domains [40]. The bands observed near 1160 cm^−1^ and 1210 cm^−1^ were assigned to the –C–O–C– stretching of the ester linkages, while the absorption at about 1044 cm^−1^ corresponded to the –O–C–C– stretching vibration. Moreover, the band around 956 cm^−1^ was linked to the bending vibration of the –C–OH group from terminal carboxylic acids. Upon incorporation of HSO, the FTIR spectrum of the PBS-HSO blend preserved the main spectral features of PBS, suggesting that the polymer backbone remained largely intact. Nonetheless, minor spectral modifications were detected. A shoulder emerging around 3010 cm^−1^ associated with the =C–H stretching of vinyl groups in unsaturated fatty acids, pointed to the presence of double bonds introduced by the oil. In the carbonyl region of the FTIR spectra (Figure 4b), a distinct broadening of the carbonyl stretching region associated with the amorphous phase (around 1720–1730 cm^−1^) was observed in the PBS-HSO sample (red curve) compared to neat PBS (black curve). This outcome was expected, as hemp oil acts as a plasticizer, promoting intermolecular mobility of the amorphous fraction of the carbonyl region, in this way highlighting both its active role in modifying the local environment of the ester carbonyl groups and its physical interaction with the polymer matrix.

#### 3.2.4. Differential Scanning Calorimetry (DSC)

**Figure 5:**
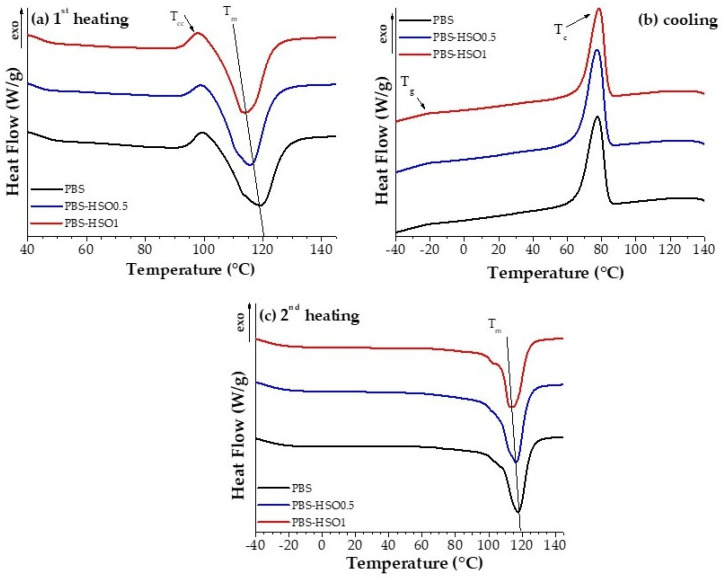
DSC thermograms of PBS and PBS-HSO blends with different amounts of HSO obtained by 2.5 min of MAE: (**a**) 1st heating run (**b**) cooling run, and (**c**) 2nd heating run. All of the experiments were performed at 20 °C/min.

The DSC thermogram of the PBS film is reported in Figure 5 and the corresponding thermal parameters (T_m1_, T_m2_, T_c_, T_g_, ΔH_m_, and χ_c_) are listed in Table 2.

During the 1st heating run of the PBS film, it was possible to observe a weak endothermic peak at around 99.4 °C (T_cc_) that preceded the main melting temperature at 119 °C (T_m_), as typically reported for PBS [41]. The broad melting peak of PBS highlighted a wider distribution in the size of the crystals formed during film processing [42]. Furthermore, during the 2nd heating run, the cold crystallization phenomenon (T_cc_) was not observed. The same behavior was exhibited by the PBS-based blends. However, with the increase in the oil content in the PBS film, T_m1_ and T_cc_ in the 1st run and T_m2_ in the 2nd run slightly decreased. These results confirmed that the presence of the oil modified the crystallization ability and in turn the melting behavior, as already observed by XRD [40,43].

No substantial differences were found in the shape and temperature of the crystallization peak in the cooling run for the PBS blends with respect to neat PBS; there was only a small increase in T_c_ for the blend containing 1 wt% of HSO. Finally, the evaluation of the glass transition temperature (T_g_) from the cooling and second heating run suggested that the presence of HSO had no effect on this parameter: all of the materials analyzed showed the same T_g_, equal to −17 °C. This result aligned with some findings in the literature [26,43,44,45,46]. In fact, according to IUPAC (International Union of Pure and Applied Chemistry), a plasticizer is typically added to polymers to enhance flexibility and lower the T_g_ by increasing chain mobility and reducing intermolecular forces. However, in some cases, the expected reduction in T_g_ by DSC did not occur.

**Table 2:** Crystallinity degree, calculated by XRD, and thermal parameters, calculated by DSC, of PBS and blend films containing different amounts of HSO. T_g_ was extracted from the first derivative curve of the cooling thermogram. ΔH_0_ of 100% crystalline PBS = 110.3 J/g [28].

		1st Heating				Cooling			2nd Heating		
	χ_c xrd_ (%)	T_cc_ (°C)	T_m1_ (°C)	ΔH_m1_ (J/g)	χ_c1_ (%)	T_c_ (°C)	ΔH_c_ (J/g)	T_g_ (°C)	T_m2_ (°C)	ΔH_m2_ (J/g)	χ_c2_ (%)
PBS film	51	99.4	119.0	54.9	49.7	77.5	65.6	−17.0	117.6	54.4	49.3
PBS 0.5%	45	99.1	117.9	54.1	49.3	77.5	73.2	−17.0	117.2	54.0	48.5
PBS 1%	44	98.9	113.6	50.2	45.9	78.4	61.3	−17.0	114.5	51.2	46.9

#### 3.2.5. Thermogravimetric Analyses (TGA)

**Figure 6:**
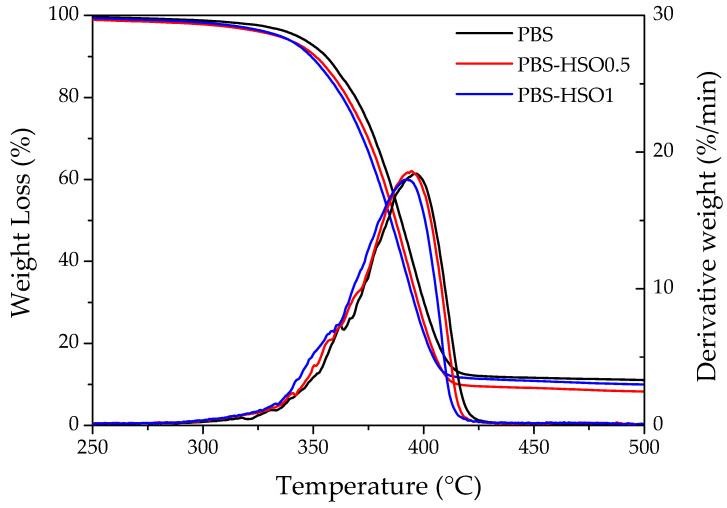
TGA and DTG thermograms recorded at 10 °C min^−1^ in a nitrogen atmosphere for PBS and PBS-HSO blends.

The TGA thermograms are shown in Figure 6. Although the degradative profiles of the PBS and PBS/oil films were quite similar, the T_onset_ and T_max_ values of the PBS/oil films were noticeably lower than those of pure PBS, as evidenced in Table 3. In particular, PBS-HSO0.5 and PBS-HSO1 exhibited T_onset_ and T_max_ values approximately 23 °C and 5–6 °C lower than those of neat PBS, respectively. This evidence suggested that the presence of extracted HSO slightly reduced the thermal stability of the blend films with respect to the PBS film.

These results could be due to HSO intercalating among polymer chains, working as a PBS thermal pro-degrading agent, thereby inducing an increase in macromolecular mobility in this way encouraging PLA thermal degradation [47]. However, the thermal stability, although slightly reduced by the addition of oil, still allowed these materials to be safely used in the packaging field, where high temperatures are not required [48]. In more detail, the required thermal stability of polymeric films for real-world packaging depends on the intended application. The higher temperature range is up to 120–130 °C, required for microwaveable and boil-in-bag packaging [49], or 115–135 °C for retort applications [50]. The PBS-HSO blends were stable up to ~300 °C, which is more than suitable for food packaging applications since typical food packaging scenarios involve much lower temperatures.

#### 3.2.6. Mechanical Properties

**Figure 7:**
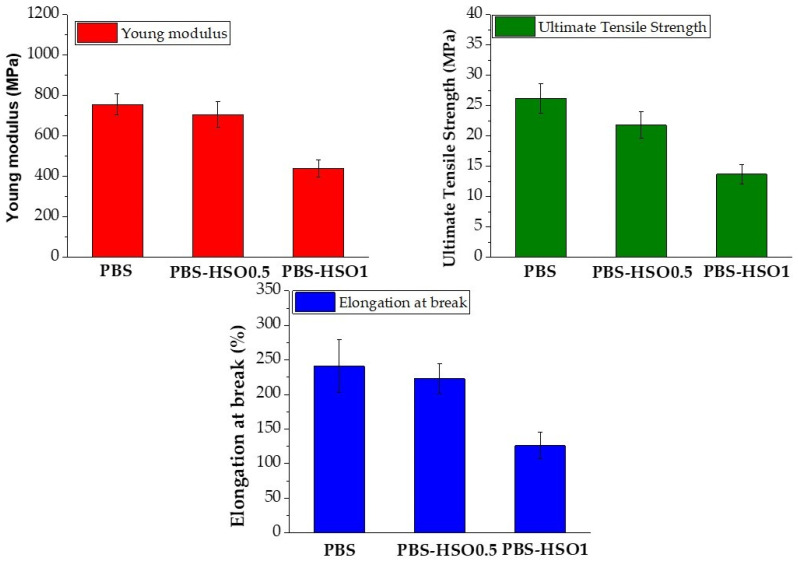
Young’s modulus (MPa), ultimate elongation at break (MPa). and elongation at break (%) for PBS and PBS-HSO films.

Figure 7 displays the mechanical parameters of the PBS-HSO films. The Young’s modulus, tensile strength, and elongation at break were found to decrease in both doped compositions. In more detail, for PBS-HSO0.5, they decreased by approximately 16%, 7%, and 7%, respectively, with respect to PBS. More significant were the reductions observed for PBS-HSO1, which showed a Young modulus of 440 MPa (−42%), an elongation at break of 126% (−47%), and an ultimate strength of 14 MPa (−47%). The presence of HSO reduced the intermolecular forces and rigidity of PBS, facilitating the disentanglement of PBS molecules, thus causing decreases in tensile strength and Young’s modulus. Additionally, the non-uniform dispersion of the oil within the polymer matrix may have introduced defects, further compromising the elongation at break; as a result, a decreasing of the polymer chain extensibility and strain at break was observed [51].

#### 3.2.7. Migration Tests in Food Simulants—Mass Loss

**Figure 8:**
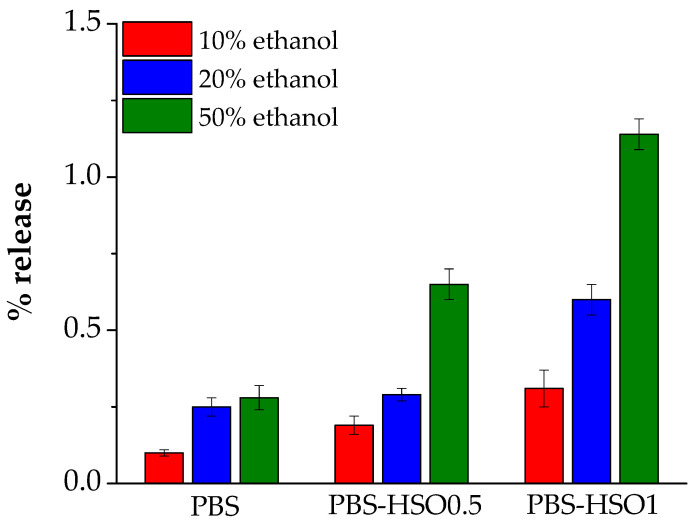
Comparison of the percentage of release of PBS and PBS-HSO blends into various food simulants.

The percentage of release (or migration) from PBS food contact material into a food simulant, calculated using Equation (4), is shown in Figure 8.

The PBS film released some substances into all of the analyzed simulants. This behavior is well-documented for neat polymers [52] and is generally attributed to migration of oligomers and/or degraded polymer chains resulting from processing preparation [53]. The amounts of substances migrating from PBS into foodstuffs increased with the increase in the “fat” character of the simulant. This was primarily due to the higher solubility of migrating organic compounds in fat than in water, rather than an increase in the diffusion coefficient caused by interactions between the fat and plastic, as is commonly assumed [52]. However, as expected, from the viewpoint of safety, the migration values of PBS were below the regulated limit of 60 mg/kg of simulant (i.e., 10, 23, and 27 mg/kg for 10, 20, and 50% ethanol simulant, respectively). For the blends, the values of migration increased with respect to those of the neat polymer due to the presence of oil. Moreover, the values rose with the increase in the oil content and with the “fat” character of the simulant. For the composites PBS-HSO0.5 and PBS-HSO1, the migration values were still below the limit for water-based simulants (i.e., 10 and 20% ethanol), but they became 65 and 105 mg/kg when 50% ethanol simulant was used. This indicated that the blend films were not suitable for food with lipophilic properties, but they can be used for aqueous, acidic, and alcoholic foods. It worthwhile to underline that these results offer a preliminary assessment of the safety of these materials. Testing on real food is mandatory for obtaining more accurate, relevant, and reliable data.

## 4. Conclusions

This study compares microwave-assisted extraction (MAE) and Soxhlet extraction using n-hexane to recover oil from hemp seeds, evaluating the yield, composition, and antioxidant activity. MAE proved highly efficient, achieving similar results to a 6 h Soxhlet extraction in just 2.5 min, demonstrating a promising, energy-efficient alternative for oil recovery from hemp seeds. The extracted hemp seed oil (HSO) was incorporated at 0.5, 1, and 2.5 wt% into poly (butylene succinate) (PBS) films for food packaging via compression molding. Due to oil exudation, the 2.5 wt% blend was excluded from further testing. The SEM analysis showed good oil dispersion and compatibility. The FTIR analysis revealed broadening of the carbonyl stretching region (1720–1730 cm^−1^) in the PBS-HSO films, indicating enhanced intermolecular mobility in the amorphous phase due to the oil’s plasticizing effect. Furthermore, the incorporation of HSO resulted in a decrease in crystallinity by 14% and thermal parameters (i.e., T_m1_ and T_cc_ in the first DSC run and T_m2_ in the second run), attributed to the oil’s interference with the organization and crystallization of PBS chains. However, the expected reduction in T_g_ was not observed. Mechanical properties decreased at both oil concentrations, with PBS-HSO1 showing the most significant reductions: Young’s modulus dropped to 440 MPa (−42%), elongation at break to 126% (−47%), and tensile strength to 14 MPa (−47%). These effects were attributed to weakened intermolecular interactions and enhanced molecular disentanglement caused by the oil. Finally, the migration tests in food simulants revealed that the blend films were not suitable for foods with lipophilic properties (migration values of 65 and 105 mg/kg for PBS-HSO0.5 and PBS-HSO1, respectively), but they can be used for aqueous, acidic, and alcoholic foods. The incorporation of hemp seed oil into a PBS film supports the development of bio-based biodegradable food packaging materials with tailored flexibility and thermal properties.

## Figures and Tables

**Table 1:** Extraction yields and DPPH values for HSO extracted by MAE and SOT at different times.

Extraction Method	Time of Extraction (min)	Yield (%)	DPPH (%)	
			Initial *	After thermal treatment **
MAE	2.5	27 ± 2	67 ± 3	68 ± 2
MAE	5	26 ± 1	68 ± 2	67 ± 1
MAE	15	26 ± 1	71 ± 2	71 ± 3
MAE	30	30 ± 2	68 ± 1	69 ± 1
MAE	60	30 ± 3	68 ± 2	69 ± 2
SOT	360	29 ± 3	68 ± 1	68 ± 1

* Values obtained after oil extraction by MAE and SOT. ** Values obtained after oil heating from 25 to 140 °C at 10 °C/min and kept at 140 °C for 20 min.

**Table 3:** Thermal parameters, determined by TGA, of PBS-based films.

Sample	T_onset_ (°C)	T_peak_ (°C)
PBS	324.7	397.2
PBS-HSO0.5	301.3	393.3
PBS-HSO1	301.5	391.5

## Data Availability

The raw data supporting the conclusions of this article will be made available by the authors upon request.
